# Paradigmatic De Novo *GRIN1* Variants Recapitulate Pathophysiological Mechanisms Underlying GRIN1-Related Disorder Clinical Spectrum

**DOI:** 10.3390/ijms222312656

**Published:** 2021-11-23

**Authors:** Ana Santos-Gómez, Federico Miguez-Cabello, Natalia Juliá-Palacios, Deyanira García-Navas, Víctor Soto-Insuga, Juan J. García-Peñas, Patricia Fuentes, Salvador Ibáñez-Micó, Laura Cuesta, Ramón Cancho, Patricia Andreo-Lillo, Gema Gutiérrez-Aguilar, Olga Alonso-Luengo, Ignacio Málaga, Antonio Hedrera-Fernández, Àngels García-Cazorla, David Soto, Mireia Olivella, Xavier Altafaj

**Affiliations:** 1 Neurophysiology Laboratory, Department of Biomedicine, Faculty of Medicine and Health Sciences, Institute of Neurosciences, University of Barcelona, 08036 Barcelona, Spain; asantos@ub.edu (A.S.-G.); fj.miguez3@gmail.com (F.M.-C.); davidsoto@ub.edu (D.S.); 2 August Pi i Sunyer Biomedical Research Institute (IDIBAPS), University of Barcelona, 08036 Barcelona, Spain; 3 Neurometabolic Unit, Department of Neurology, Hospital Sant Joan de Déu—CIBERER, 08950 Barcelona, Spain; njulia@sjdhospitalbarcelona.org (N.J.-P.); agarcia@sjdhospitalbarcelona.org (À.G.-C.); 4 Department of Pediatric Neurology, Hospital Universitario San Pedro de Alcántara, 10001 Cáceres, Spain; deya-nira121@hotmail.com; 5 Neurology Service, Hospital Niño Jesús, 28009 Madrid, Spain; victorsotoinsuga@gmail.com (V.S.-I.); jgarciadelarape.1961@gmail.com (J.J.G.-P.); 6 Neuropediatrics Unit, Hospital Clínico Santiago de Compostela, 15706 Santiago de Compostela, Spain; Patricia.Fuentes.Pita@sergas.es; 7 Pediatric Neurology Unit, Arrixaca Universitary Hospital, 30120 Murcia, Spain; salibmi@hotmail.com; 8 Department of Paediatrics Neurology, Hospital de Manises, 46940 Valencia, Spain; lcuesta@hospitalmanises.es; 9 Pediatric Neurology, Department of Pediatrics, Hospital Universitario Río Hortega, 47012 Valladolid, Spain; rcanchoc@saludcastillayleon.es; 10 Neuropediatric Unit, Pediatric Department, University Hospital of Sant Joan d’Alacant, 03550 Sant Joan d’Alacant, Spain; andreo_pat@gva.es; 11 Pediatrics Unit, Hospital Universitario de Jerez de la Frontera, 11407 Jerez de la Frontera, Spain; gemamegga@gmail.com; 12 Department of Pediatrics, Hospital Universitario Virgen del Rocío, 41013 Sevilla, Spain; oaluengo@gmail.com; 13 Child Neurology Unit, Pediatrics Department, Hospital Universitario Central de Asturias, 33011 Oviedo, Spain; neuropediatria.huca@gmail.com (I.M.); antonio.hedreraf@sespa.es (A.H.-F.); 14 Bioinfomatics and Medical Statistics Group, University of Vic—Central University of Catalonia, 08500 Vic, Spain

**Keywords:** GRIN-related disorders, glutamatergic neurotransmission, NMDA receptors, neurodevelopmental disorders

## Abstract

Background: GRIN-related disorders (GRD), the so-called grinpathies, is a group of rare encephalopathies caused by mutations affecting *GRIN* genes (mostly *GRIN1*, *GRIN2A* and *GRIN2B* genes), which encode for the GluN subunit of the *N*-methyl D-aspartate (NMDA) type ionotropic glutamate receptors. A growing number of functional studies indicate that GRIN-encoded GluN1 subunit disturbances can be dichotomically classified into gain- and loss-of-function, although intermediate complex scenarios are often present. Methods: In this study, we aimed to delineate the structural and functional alterations of *GRIN1* disease-associated variants, and their correlations with clinical symptoms in a Spanish cohort of 15 paediatric encephalopathy patients harbouring these variants. Results: Patients harbouring *GRIN1* disease-associated variants have been clinically deeply-phenotyped. Further, using computational and in vitro approaches, we identified different critical checkpoints affecting GluN1 biogenesis (protein stability, subunit assembly and surface trafficking) and/or NMDAR biophysical properties, and their association with GRD clinical symptoms. Conclusions: Our findings show a strong correlation between *GRIN1* variants-associated structural and functional outcomes. This structural-functional stratification provides relevant insights of genotype-phenotype association, contributing to future precision medicine of GRIN1-related encephalopathies.

## 1. Introduction

Neurodevelopmental disorders (NDDs) result from the disturbance of critical neurodevelopmental processes, caused by the presence of genetic alterations. Besides the identification of several genes associated with NDDs, the environmental factors can also influence the clinical outcomes associated with NDDs. Recently, next-generation sequencing (NGS) technologies allowed the identification of monogenic causes of NDD, with several genes converging on the glutamatergic synapse [1]. Disease-associated variants of *GRIN* genes (e.g., mostly affecting *GRIN1*, *GRIN2A*, *GRIN2B* and *GRIN2D)*, which encode *N*-methyl-D-aspartate (NMDA) receptor subunits and oligomerize to form ionotropic glutamate receptors, have been recently associated with rare neurodevelopmental encephalopathies [2,3,4]. Further, the growing number of *GRIN* variants associated with neurodevelopmental encephalopathies, followed by functional and clinical studies coined the gene-based classification of this group of genetic neurodevelopmental disorders, namely *GRIN* genes-related Disorders (GRD), the so-called grinpathies. GRD is a group of rare paediatric encephalopathies (estimated prevalence 1:5000 births; [5]) with an autosomal dominant inheritance pattern [6,7,8,9]. The heterogeneous clinical spectrum of GRD englobes developmental delay with hypotonia, intellectual disability (ID), autism spectrum disorder (ASD) traits and epilepsy [6,10].

NMDA receptors belong to the ionotropic glutamate receptors family, playing a pivotal role in glutamatergic neurotransmission. NMDA receptors are heterotetrameric ligand-gated ion channels composed by two obligatory GluN1 subunits, and a combination of two GluN2(A–D) and/or GluN3(A,B) subunits [11]. To exert their key neuronal function, NMDARs need to be finely controlled, both in terms of gene expression (under a precise spatio-temporal pattern), subcellular location, surface density and biophysical activity [12]. Concomitantly, genetic variants affecting these different biochemical, cellular, and physiological checkpoints can impact NMDAR function, ultimately leading to neuronal activity dysfunction, abnormal brain wiring and neurological symptoms. Tetrameric NMDAR architecture consists of an amino terminal domain (ATD), a ligand binding domain (LBD, binding the coagonists glutamate and glycine/D-serine), a transmembrane domain (TMD) and a large carboxy terminal domain (CTD) with non-resolved structure. Functionally, concurrent binding of glycine/D-serine and glutamate to GluN1 and GluN2 subunits, respectively, are required for receptor activation [13] and induce NMDAR conformational changes [14,15].

Along the last years, many efforts have been focused to address the functional substrate of GRD, namely the identification of *GRIN* variants-related alterations of the NMDAR function. As for other monogenic and polygenic channelopathies, mutations affecting *GRIN* genes have been roughly dichotomically classified in two opposite gain of function (GoF) and loss of function (LoF). This functional stratification allowed to primarily split *GRIN* de novo variants (*GRIN*-DNV) in two main categories, with concomitant valuable therapeutic outcomes (e.g., decision-making of personalised therapeutic strategies) [16,17]. Nevertheless, both from a theoretical and experimental point of view, the growing number of functionally annotated *GRIN* variants suggest that rather than a dichotomic scenario, it can be hypothesised that *GRIN* variants repertoire covers a continuum of functional outcomes underlying GRD clinical spectrum.

In order to evaluate this hypothesis, we have characterised the structural and functional outcomes of novel *GRIN1*-DNVs, tentatively annotated as disease-associated and likely disturbing NMDARs activity. These variants have been comprehensively annotated by means of molecular modeling, followed by biochemistry, cell biology, computational and electrophysiological experimental approaches. The multidisciplinary annotation of *GRIN1*-DNV confirmed the presence of multiple and complex *GRIN1*-DNV-related NMDAR dysfunctional fingerprints, supporting the *GRIN*-DNV continuum hypothesis. Further, *GRIN1*-DNV variants were correlated with clinically deep-phenotyped *GRIN1*-DNV patients cohort, revealing an association between *GRIN1*-DNVs functional categories and GRD severity.

## 2. Results

### 2.1. GRIN1 Variants-Associated Clinical Cases

In the last decade, next-generation sequencing implementation to clinical diagnosis has emerged and allowed reporting of a growing number of both neutral and disease-associated *GRIN1* variants. The integration of genotype-phenotype data allowed to define *GRIN1* genetic variants based on their differential vulnerability/resistance to trigger NMDAR-mediated glutamatergic dysfunction. Based on this domain stratification, in the present study we have selected a panel of *GRIN1* variants scattered along GluN1 subunit topological domains, ranging from relatively tolerant to highly vulnerable to genetic variation. Patients harbouring *GRIN1* disease-associated variants have been homogeneously clinically assigned (Table 1) and relative *GRIN1* variants have been functionally annotated.

Clinical assessment of *GRIN1* variants-harbouring Spanish patients has been conducted by the Spanish GRD Neuropaediatrics network. In order to define precise and consensus clinical items criteria (items, severity scores), in the absence of an available GRIN1-related disorders natural history registry, the most prevalently reported GRIN1-related symptoms [6,9] have been included. Therefore, clinically diagnosed intellectual disability (ID), communication deficits, social interaction defects, tone and motor impairment, seizures, and gastro-intestinal distress, have been included (Table 1).

In agreement with the constitutive presence of GluN1 subunit in the NMDAR and the pivotal role of this glutamatergic receptor in neuronal function and brain wiring [12], individuals harbouring disease-associated *GRIN1* mutations almost invariably (14 out of 15) present severe ID, with the exception of a patient carrying *GRIN1*-M818V exhibiting moderate ID. The severity of ID is directly correlated with the absence or highly poor oral communication. In contrast with the pathognomic presence of ID and language deficits, *GRIN1*-DNVs cohort showed a variable presence of mood disorders (e.g., irritability, aggressivity, hyperactivity; 8 out of 15 individuals), as well as sleep disorder (6 out of 15), in a variant-dependent manner. Similarly, despite the presence of severe verbal communication deficits, *GRIN1*-DNV cohort exhibits a variable manifestation of autism spectrum disorders (ASD) traits (6 out of 15). In terms of motor phenotypic alterations, *GRIN1*-DNVs patients showed variable motor outcomes, ranging from mild to severe. In this cohort, 10 out of 15 patients exhibited movement disorders, including stereotypies, dystonia, akinetic rigid syndrome, hyperkinesia, oculogyric crisis and/or dyskinetic movements. Movement disorders were accompanied by the presence of heterogeneous gait alterations, ranging from severe to moderate. Normal walking was present in 6 out of 15 *GRIN1*-DNVs patients, although the acquisition was delayed compared to the neurotypical population. Alterations of muscle tone (axial hypotonia, limbs spasticity, rigidity, hypertonia) and hyperlaxity were also generally displayed in the *GRIN1*-DNV cohort (12 out of 15 cases).

Congruent with previous reports associating *GRIN1*-DNVs with epileptiform episodes and abnormal brain morphology, the study cohort also showed a high prevalence of epileptic episodes (11 out of 15) and the presence of macroscopic brain morphological abnormalities (7 out of 15 cases). The onset, type and severity of seizures was highly variable (in a variant-dependent manner), as well as antiepileptic drugs efficacies. Brain imaging data showed a spectrum of gross brain abnormalities, although no commonalities were detected and polymicrogyria was not present in the cohort. Besides the presence of the aforementioned primary central nervous system alterations, GRD individuals also display systemic disturbances. Notably, the presence of gastro-intestinal (GI) distress has been reported by families and caregivers. The study of the *GRIN1*-DNV cohort showed a relative high prevalence of GI distress (5 out of 15), mostly associated with dysphagia, gastroesophageal reflux, and constipation.

Overall, deep-phenotyping of *GRIN1*-DNV cohort showed the presence of pathognomonic symptoms (ID, motor phenotypes), together with a spectrum of variable clinical alterations dictated in a variant-dependent manner potentially allowing to correlate genotype-phenotype severity stratification.

### 2.2. Molecular Modeling of GRIN1-DNVs in Triheteromeric NMDA Receptors

**Figure 1:**
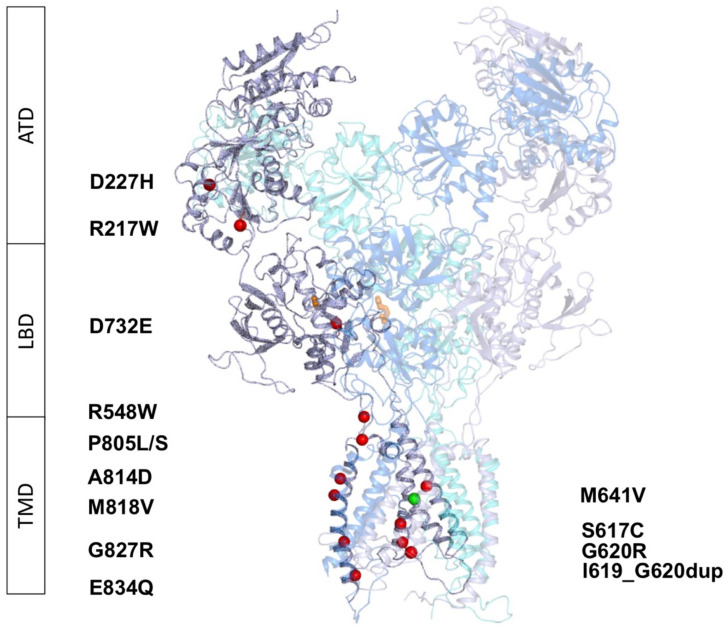
Distribution of de novo *GRIN1* variants along GluN1 subunit domains and NMDAR structure, composed of two GluN1 (in grey and purple), one GluN2A (cyan), and one GluN2B (blue) subunits. Variants are shown as red spheres, agonist and coagonists in orange and Mg^2+^ as a green sphere. ATD: Amino Terminal Domain, LBD: Ligand Binding Domain, TMD: Transmembrane Domain.

In order to predict the potential impact of *GRIN1* variants on the NMDA receptor structure and to infer the putative functional outcomes, the molecular models of *GRIN1*-DNV containing (GluN1-DNV)_2_-GluN2A-GluN2B triheteromeric NMDA receptors were generated. The models were based on X-ray crystal structures of the subunits, reconstruction of the undetermined regions of the receptor and Energy Minimization. Figure 1 shows the distribution of the GluN1 variants, with two variants located within the amino terminal domain (ATD), one variant at the ligand binding domain (LBD) and ten variants at the transmembrane domain (TMD).

#### 2.2.1. Variants Affecting GluN1 Subunit Folding and/or NMDAR Oligomerisation

**Figure 2:**
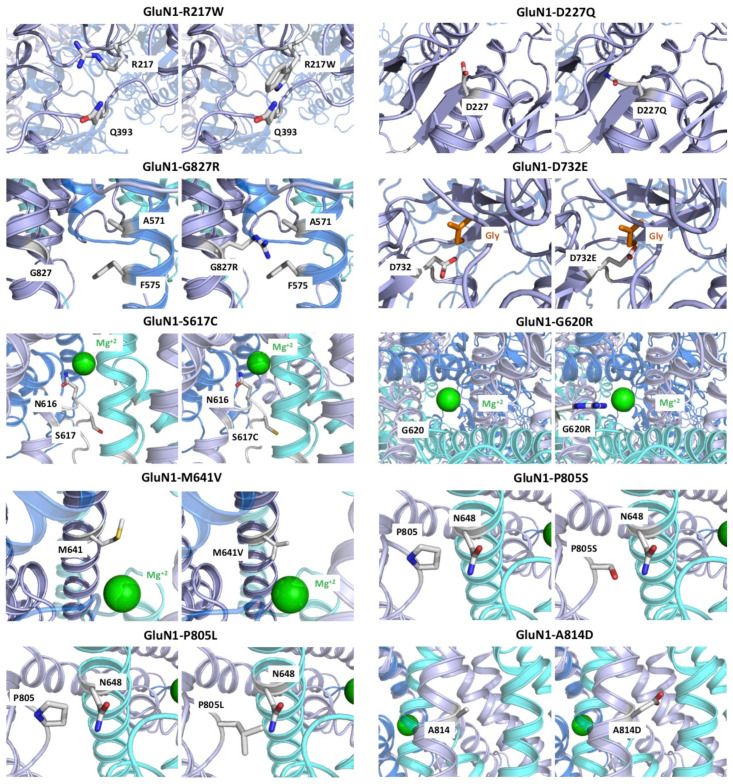
NMDAR structure, composed of two GluN1 subunits (in grey and purple), one GluN2A (cyan), and one GluN2B (blue) subunit. Variants R217W, D227H, G827R and E834Q affect the folding of the protein. D732E variant avoids the binding of the coagonist glycine. S617C, M641, G620R, P805L, P805S, A814D and M181V affect the channel properties. Glycine is shown in orange and magnesium is shown in green.

According to the molecular models, *GRIN1* variants R217W, D227Q, G827R, E834Q and I619_G620dup, avoid proper protein folding and, consequently, lead to misfolded proteins and/or NMDARs that are unable to dock the plasma membrane (Figure 2). *GRIN1*-R217 is located in a loop at the lower lobe of the ATD and the substitution of an arginine by a tryptophane residue is predicted to provoke a steric clash with Q393, located in a contiguous loop, avoiding the folding of the GluN1 subunit. *GRIN1*-D227 residue is situated in an alpha helix at the lower lobe of the ATD, pointing towards a loop. Missense mutation to a histidine residue (D227H) induces a steric clash with this loop due to a longer side chain, avoiding protein folding. *GRIN1*-G827 amino acid is located at the transmembrane domain M4, close to the intracellular domain. Mutation from a glycine (without side chains) to an arginine residue (*GRIN1*-G827R, containing a big positive amino acid side chain) results in a steric clash with the M1 transmembrane domains from GluN2B/GluN2A subunits, completely abolishing NMDAR oligomerisation. *GRIN1*-E834 amino acid is located at the limit between the transmembrane domain and the intracellular domain. The amino acid substitution *GRIN1*-E834Q implies the loss of a negative charge and is predicted to affect NMDAR anchoring to the plasma membrane. Last, *GRIN1*-I619_G620dup implies an insertion of two residues at the TMD, completely avoiding protein folding. In summary, these five *GRIN1*-DNVs induce a change in the amino acid charge that produce a drastic change of the physicochemical properties of the amino acids which ultimately are predicted to severely compromise NMDAR assembly.

#### 2.2.2. GRIN1-D732E Missense Variant Affects Coagonist Binding

*GRIN1*-D732 residue is involved in glycine binding at the LBD. Mutation to a glutamate residue in the D732E variant introduces a longer side chain that would, potentially, impair glycine/D-serine binding. This substitution would therefore putatively cause an hypofunctionality of *GRIN1*-D732E subunit-containing NMDARs, in the presence of non-saturating glycine/D-serine concentration within the synaptic cleft. Since coagonists concentrations are saturating in our experimental design, this hypothesis has not been evaluated in the electrophysiological studies (Section 2.3.2) and the agonists potency alterations remain elusive.

#### 2.2.3. GRIN1-DNVs Affecting NMDAR Channel Properties

GluN1-R548 residue is located at the linker between the LBD and the TM domain. Mutation to tryptophan affects the signal transduction across the LBD and the TMD. The amino acid residue GluN1-S617 is located within the NMDAR channel pore, at the vicinity of GluN1-N616 residue that interacts with Mg^2+^ (NMDAR blocker at resting membrane potential). Despite the substitution to a cysteine residue in GluN1-S617C only implies a minor change in the physicochemical properties of the side chain, molecular modeling indicates a modification of the magnesium cavity and the pore channel. GluN1-G620 residue is placed at the bottom of the channel, exposed to the pore channel. Mutation to arginine in GluN1-G620R introduces a big positive amino acid side chain to the center of the pore channel, affecting ion influx and avoiding magnesium block. GluN1-M641 amino acid is located at the pore of the channel (M3 domain) and close to Mg^2+^. GluN1-M641V variant results in a positioning of V641 to the pore channel, facing magnesium. Thus, GluN1-M641V variant is predicted to affect magnesium-mediated NMDAR blockade. GluN1-P805 is situated within a loop at the beginning of M4 pointing the SYTANLAAF motif of GluN2A,B subunits. Mutation to leucine or serine (*GRIN1*-P805L and *GRIN1*-P805S, respectively) distorts the bending of M4. While leucine presence in *GRIN1*-P805L variant positions close to N648 residue of the SYTANLAAF motif, substitution to a serine residue in P805S variant is not able to reach the SYTANLAAF motif. Thus, *GRIN1*-P805L is predicted to induce a higher distortion and, consequently, functional, and clinical outcomes, than *GRIN1*-P805S. *GRIN1*-A814 amino acid is located at the transmembrane domain M4, facing the membrane and M2 domain of GluN2A/GluN2B subunits. Mutation to an aspartate residue in *GRIN1*-A814D is predicted to affect subunit interaction due to a new negative charge between the two subunits. GluN1-M818 is located within the M4 domain of the TMD, facing the cell membrane. In addition to this location, the substitution to a valine residue (*GRIN1*-M818V variant) would potentially induce a slight change, since the side chain physicochemical properties are similar, and the most significant difference is the introduction of a bigger side chain. Overall, the structural analysis of *GRIN1* variants affecting NMDAR channel gating showed that *GRIN1*-P805L, -G620R and -M641V are potentially causing a severe effect on the structure and function on the NMDAR, *GRIN1*-S617C, -P805S and -M818V are predicted as moderate, while *GRIN1*-A814 is predicted as causing weak disturbance of the NMDAR.

### 2.3. Functional Annotations of GRIN1-DNVs

#### 2.3.1. *GRIN1* Variants Affecting NMDAR Surface Density

In order to identify the potential pathophysiological alterations resulting from likely pathogenic *GRIN1* de novo variants present in GRD patients, the mutations of interest were introduced by site-directed mutagenesis in *GRIN1* expression plasmids. Further, the potential alterations of either NMDAR biogenesis or NMDAR gating properties were evaluated.

GluN subunits of the NMDAR are synthesized in the endoplasmic reticulum (ER), where translation and particular post-translational modifications are achieved. Ultimately, these ER-regulated mechanisms allow GluN subunits dimerisation, oligomerisation and further trafficking to the plasma membrane [11,18]. To characterize potential disturbance of ER-related control of NMDAR abundance, mutant GluN1 subunits protein stability was evaluated. HEK-293T cells were transiently transfected with wildtype *GRIN2B* and *GRIN1* (wildtype and mutant) constructs, and protein extracts were analysed by western blot. While most of *GRIN1* missense variants did not affect GluN1 subunit stability, *GRIN1*-R217W immunodetection was negligible, indicating unstable or nonexistent GluN1-R217W subunit (data not shown) that might be a primary cause of NMDAR surface density decrease and NMDAR hypofunctionality.

**Figure 3:**
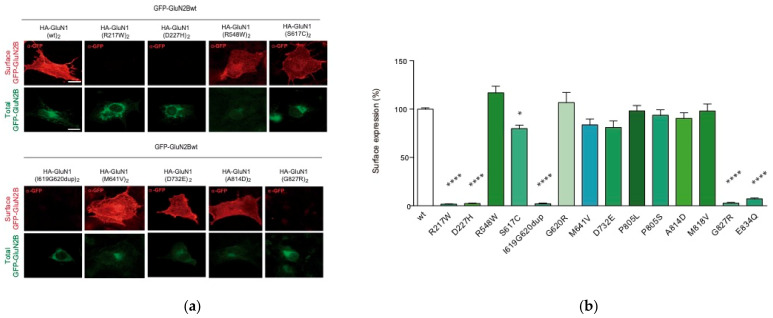
Effects of biallelic *GRIN1*-DNVs on NMDAR surface expression. (**a**) Immunofluorescence analysis of NMDAR surface expression in COS-7 cell line co-transfected with GFP-*Grin2b* and HA-*Grin1* (wildtype and/or mutant) constructs; scale bar: 10 μm; (**b**) Bar graph representing the mean ± SEM of cell surface expression of NMDAR (N = 16–38 cells per condition, from 3 to 4 independent experiments; ns *p*-value > 0.05; * *p*-value < 0.05; **** *p*-value < 0.0001, one-way ANOVA with Bonferroni’s post hoc test).

In order to exert their function, NMDAR surface expression and subcellular location need to be finely tuned. Concomitantly, any disturbance affecting NMDAR density and/or subcellular distribution might impair NMDAR-mediated signaling. According to these intrinsic properties, we explored surface density of NMDARs harbouring the selected *GRIN1* variants present in the cohort of paediatric patients. Immunofluorescence analysis of NMDAR surface expression was conducted in COS-7 cells transiently co-transfected with *GRIN2B*wt and biallelic *GRIN1* variants constructs. Immunofluorescence analysis showed that *GRIN1*-G827R, *GRIN1*-E834Q and *GRIN1*-I619_G620dup variants abolished surface expression of the mutant subunit, while the other *GRIN1* variants evaluated do not compromised NMDAR delivery to the cell surface (Figure 3), indicating that these variants do neither alter the oligomerization nor the delivery of mutant subunit-containing NMDARs.

**Figure 4:**
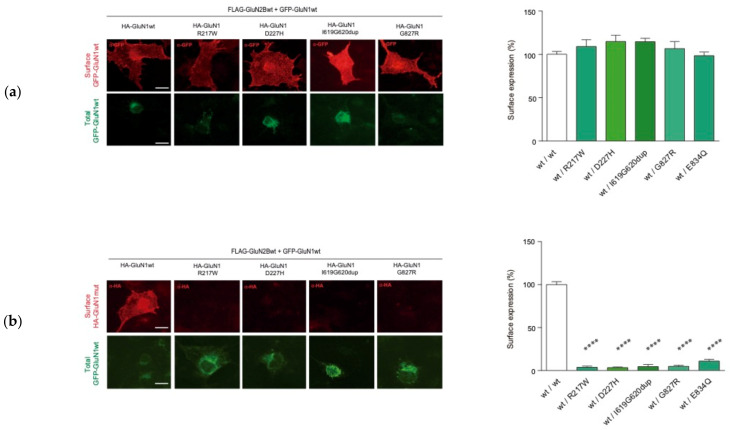
Analysis of intermolecular GluN1 wildtype and mutant interaction effects on NMDAR surface expression. (**a**) *Left*, Representative image of immunofluorescence analysis of GFP-GluN1-wt surface expression in COS-7 cell line co-transfected with GFP-*GRIN*1-wt, HA-*GRIN1*-DNV and Flag-GRIN2B-wt; scale bar: 10 μm; *Right*, Bar graphs representing the mean ± SEM of cell surface expression of NMDAR (N = 16–38 cells per condition, from 3–4 independent experiments; ns, *p*-value > 0.05; **** *p*-value < 0.0001, one-way ANOVA with Bonferroni’s post hoc test). (**b**) *Left*, Representative image of immunofluorescence analysis of HA-GluN1(wt/mutant) surface expression in COS-7 cell line co-transfected with GFP-*GRIN1*-wt, HA-*GRIN1*-DNV and Flag-GRIN2B-wt; scale bar: 10 μm; *Right*, Bar graphs representing the mean ± SEM of cell surface expression of NMDAR (N = 16–38 cells per condition, from 3 to 4 independent experiments; ns *p*-value > 0.05; **** *p*-value < 0.0001, one-way ANOVA with Bonferroni’s post hoc test).

Upon the identification of biallelic *GRIN1* de novo variants disturbing NMDAR surface density, namely *GRIN1*-SDD (surface density deficient *GRIN1* variants), we investigated the functional relationship between GluN1-SDD and GluN1wt subunits, to determine putative dominant negative or rescue effects. To test these potential effects, COS-7 cells were cotransfected with Flag-*GRIN2B*-wt construct and an equimolar ratio of differentially-tagged GFP-*GRIN1*wt and HA-*GRIN1*-SDD plasmids. Surface expression analysis showed that GluN1wt subunit surface expression was unaffected by the presence of GluN1-SDD variants, indicating that GluN1-SDD subunits do not exert a dominant negative effect over GluN1wt subunits (Figure 4a), as confirmed by electrophysiological recordings (Supplementary Materials Figure S1). In parallel with this analysis, immunofluorescence studies revealed that the presence of GluN1wt subunit was not rescuing the surface expression of GluN1-SDD subunits (Figure 4b).

*GRIN1* variants defective on cell surface expression are scattered along two main regions, namely the M4-CTD region and the ATD (amino acid substitution affecting residues at position 217 or 227). Regarding the latter, despite genotype-phenotype studies showed a low vulnerability (disease-association) of the ATD region [6], some discrete de novo missense variants have been associated with NDDs. Based on the topological organisation of the NMDAR domains, we hypothesized that *GRIN1*-ATD variants could not directly affect agonists affinities and/or gating properties of the channel, and their potential impact on NMDAR allosteric modulation shall be likely non-pathogenic. Therefore, we hypothesised that pathogenic *GRIN1*-ATD variants should result from disturbed NMDAR surface density. Thus, we selected GRIN1-ATD likely pathogenic variants previously reported (*GRIN1*-R217W and *GRIN1*-D227H; [6]). Immunofluorescence analysis was performed in COS-7 cells co-transfected with biallelic *GRIN1*-R217W or *GRIN1*-D227H (homozygous condition) and GRIN2B-wt. This analysis showed that NMDARs containing two *GRIN1* variants (homozygous situation) result on a drastic reduction of NMDARs surface expression (1.7 ± 0.5 (N = 33) and 3.2 ± 0.8 (N = 25) vs. 100 ± 1.4 (N = 236), for *GRIN1*-R217W, *GRIN1*-D227H and GRIN1-wt, respectively; *p* < 0.0001; Figure 3). Overall, these data indicate that, in addition to protein stability disturbance, *GRIN1* variants can alter receptor biogenesis and likely cause NMDAR loss of function underlying GRD. Similar to *GRIN1*-SDD variants from the patient cohort, heterozygous *GRIN1*-wt/R217W- and *GRIN1*-wt/D227H-subunit containing NMDARs did neither affect GluN1-wt subunits surface expression nor promote gross biophysical alterations of the NMDAR (Figure 4 and Supplementary Materials Figure S1).

#### 2.3.2. *GRIN1* Variants Disturbing NMDAR Channel Gating Properties

From the initial patient cohort, surface expression studies allowed to identify *GRIN1*-DNV compromising NMDAR surface density (4 out of 17) and causing a net loss-of-function with non-dominant effect over GluN1 subunits-containing NMDARs. Further, structural analysis allowed to predict putative biophysical alterations of NMDARs containing non-STD *GRIN1* variants.

**Figure 5:**
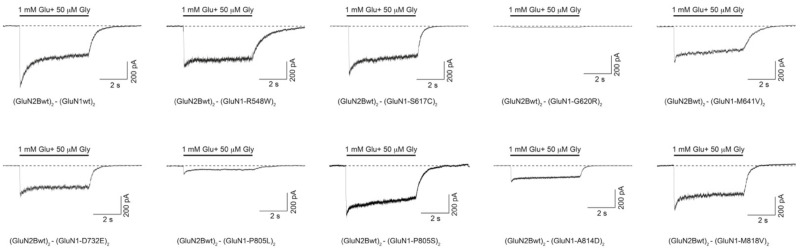

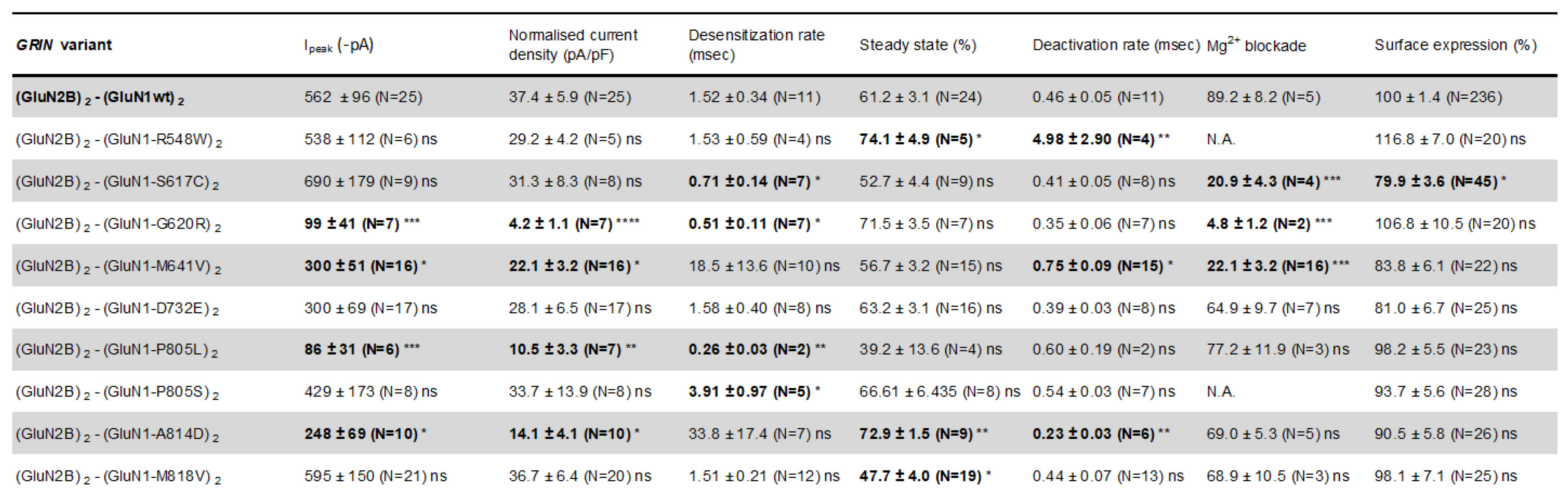
Electrophysiological characterisation of NMDAR-mediated currents in HEK-293T heterologously expressing disease-associated *GRIN1*-DNVs. (**Top**) Representative electrophysiological traces of NMDAR-mediated currents in HEK-293T cells transfected with biallelic *GRIN1*-DNVs (or GRIN1-wt) and *GRIN2B* and co-activaded by simultaneous 5-sec (bar) application of 50 μM glycine and 1 mM glutamate. (**Bottom**) Summary of functional annotations of GluN1-DNVs. Data representing mean ± SEM. ns non-significant statistical difference; * *p* < 0.05; ** *p* < 0.01; *** *p* < 0.001; **** *p* < 0.0001; NA, not assigned. Note: statistically significant differences in bold characters.

In order to functionally assess the putative biophysical alterations of *GRIN*-DNVs, and guided by structural *in silico* predictive studies, we conducted electrophysiological experiments in HEK-293T cells transiently cotransfected with plasmids encoding wildtype GluN2B subunit and wildtype and/or mutant GluN1 subunits. Following fast glutamate (1 mM) and glycine (50 μM) coagonists application, NMDAR-mediated currents were recorded. The analysis of normalized peak amplitude showed a significant decrease of the normalized peak amplitude in HEK-293T cells expressing biallelic GluN1-G620R, GluN1-M641V, GluN1-D732E, GluN1-P805L and GluN1-A814D variants (Figure 5), whereas the normalised peak current density was not affected in cells expressing the other *GRIN1*-DNV with non-affected surface density. Further, mutant *GRIN1*-containing NMDARs electrophysiological signature was accompanied by the characterisation of NMDAR kinetic parameters, playing a crucial role in fast excitatory neurotransmission. These parameters were quantified upon 5-sec duration jumps. Mutant GluN1 subunits-containing NMDARs showed alterations on channel activation, desensitization, steady-state current density and deactivation rates, in a *GRIN1* variant dependent-manner. In particular, with respect to wildtype receptors the desensitization rate was accelerated in GluN1-S617C- and GluN1-P805L-containing NMDARs (Figure 5), while it was strongly slowed down for GluN1-M641V- and GluN1-A814D-containing NMDARs (Figure 5). Steady state current density analysis showed a significant increase for *GRIN1*-R548W or -A814D-subunit containing NMDARs, and a decrease of *GRIN1*-M818V subunit containing NMDARs. Fast deactivation analysis of the NMDAR containing *GRIN1* variants products was analysed, revealing a mild or drastic increase of the deactivation rate in GluN1-M641V or GluN1-R548W-containing NMDARs, respectively (Figure 5). To complete the functional annotation of disease-associated *GRIN1* variants, Mg^2+^-mediated blockade of NMDARs was evaluated in mutant NMDARs containing GluN1 subunit mutations structurally-predicted to alter Mg^2+^ binding site. The percentage between the normalized peak amplitude in the absence vs. presence of Mg^2+^ showed that amino acid substitutions GluN1-S617C, GluN1-G620R and GluN1-M641V provoked a dramatic decrease of 1 mM Mg^2+^ blockade sensitivity at resting potentials (Figure 5).

### 2.4. GRIN1-DNV Functional Stratification and GRD Severity

The aforedescribed multidisciplinary annotation of *GRIN1*-DNVs (based on structural, cell biology and electrophysiological experimental approaches) provides a multifaceted *GRIN1* variants fingerprints, allowing their functional stratification. While overall *GRIN1* variants outcomes might be influenced by genetic, developmental, biochemical and environmental factors, the dominant inheritance pattern and high penetrance of *GRIN1* variants strongly support a strong genotype-function-phenotype correlation. Therefore, we integrated the experimental data using a hierarchical model reflecting NMDAR function governance. This model, based on the assumption that NMDAR activity is hierarchically controlled by surface levels of NMDAR, then by current density and kinetic parameters, was applied towards *GRIN1* variants functional stratification and further correlation studies between *GRIN1*-DNVs and clinical outcomes.

Surface expression studies showed that *GRIN1*-DNVs affecting NMDAR biogenesis almost completely abolished surface expression of *GRIN1*-SDD-containing NMDARs. Furthermore, co-expression of GluN1-SDD and GluN1-wt showed the lack of both a dominant negative and rescue effects, respectively. These data were confirmed by electrophysiological experiments, showing that co-transfection of GRIN1-wt and *GRIN1*-SDD do not alter channel gating properties of surface receptors (data not shown). Overall, assuming equivalent expression levels of wildtype and mutant *GRIN1* alleles, the data strongly suggest that heterozygous presence of *GRIN1*-SDD variants decreased cell surface functional NMDARs to 25% of normal density, according to mendelian ratios), causing a severe LoF.

**Figure 6:**
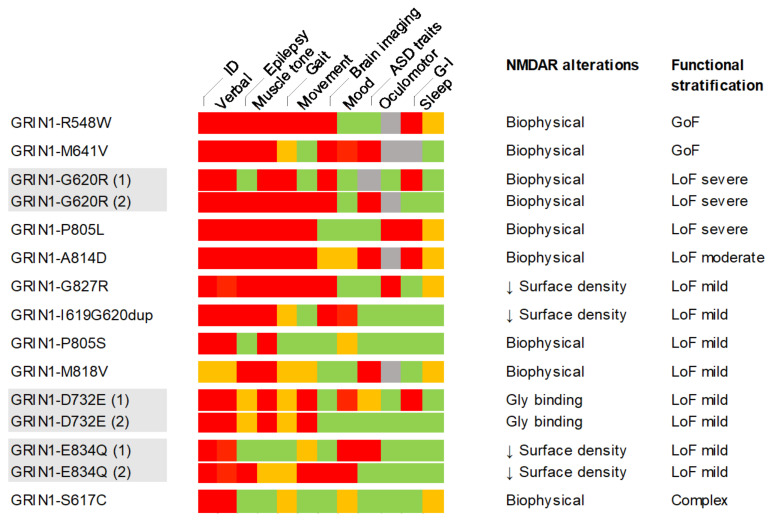
Summary of *GRIN1*-DNVs genotype-phenotype association, between severity-graded clinical symptoms and individual or functionally grouped *GRIN1* variants. *GRIN*-DNV-associated clinical symptoms (detailed in Table 1) were classified into severe (red squares), mild/moderate (orange), absent (green) or non-annotated (grey) and juxtaposed to the functional annotation categories (GoF, gain of function; LoF, loss of function).

Electrophysiological characterisation of non-SDD *GRIN1* variants showed a spectrum of biophysical alterations, in a variant-dependent manner. According to the hierarchical model, *GRIN1* variants provoking alterations of current density had an overall loss-of-function effect, partially weighted (mitigated or amplified) by alterations of kinetic parameters and Mg^2+^ blockade sensitivity. This model resulted in the classification of *GRIN1*-DNV into three main categories, namely loss-of-function, gain-of-function and complex (coexistence of loss- and gain-of-function disturbances), with a severity grade (mild/moderate/severe) (Figure 6).

The clinical assessment of individuals harbouring *GRIN1*-DNVs showed the presence of both pathognomic traits (ID, communication skills, motor alterations) of reduced clinical stratification power, together with *GRIN1* variant-dependent clinical manifestations (mood disorders, sleep pattern, GI distress) of putative clinical stratification dimension. In order to extract a clinical severity scale for further genotype-phenotype correlation, the clinical items severity (absence, mild, intermediate, severe; Figure 6) was annotated and the percentage of maximal scores was computed (Figure 6). Despite *GRIN1* cohort’s relatively small size, the heat map showed both the prevalence and severity of clinical symptoms, allowing to propose a preliminary genotype-phenotype severity stratification. Based on functional annotation studies, mutations were classified into GoF, LoF (severe, moderate, mild) and complex, and correlated with the percentage of clinical items with maximal severity score (red squares). According to non-weighted clinical severity criteria, *GRIN1* GoF and LoF (severe, moderate) showed a high severity (50% or above), while mild LoF and complex *GRIN1* variants were associated with less severe clinical symptoms (50% or below). Interestingly, *GRIN1* variants harboured by two unrelated patients (*GRIN1*-G620R, *GRIN1*-D732E, *GRIN1*-E834Q) showed similar clinical severity (same severity group), supporting the severity scale validity and in agreement with the high penetrance proposed for GRD genetic condition.

## 3. Discussion

Overall, the integrative analysis of surface expression, structural and biophysical data allowed to identify different pathophysiological mechanisms underlying GRIN1-related disorders. Despite disease-associated *GRIN* variants are traditionally dichotomically classified into loss- or gain-of-function variants, functional studies allowed to define *GRIN1* variants sub-categories based on dysfunctional NMDARs fingerprints: decreased surface expression of mutant NMDARs, disturbed current density, affected Mg^2+^ blockade and/or altered NMDAR-mediated currents kinetics. In summary, our study shows the aetiological multiplicity of disease-associated *GRIN1* variants and, together with a deep clinical phenotyping, tentatively proposes a genotype-phenotype association of GRD severity.

NMDAR functionality is a dynamic process resulting from the integration of epigenetic, genetic, environmental and cellular processes. In relation with the epigenetic control of *GRIN* genes expression (reviewed in [19]), GluN subunits have a precise spatio-temporal expression pattern that is particularly relevant in the developmental switch between GluN2B and GluN2A subunits expression [12,20]. While GluN2A-D and GluN3A,B subunits presence in the mature NMDAR is variable, GluN1 subunit is constitutively present in functional NMDAR tetramers [12]. Accordingly, mutations affecting GluN1 subunit are hypothesised to affect the ensemble of the NMDAR population, impairing glutamatergic circuitry and causing severe clinical alterations that can be modulated by synaptic activity and homeostatic processes. Deep clinical phenotyping of patients harbouring disease-associated *GRIN1*-DNV confirmed the severity of the associated clinical scenarios that cause neurodevelopmental alterations with invariable presence of intellectual disability and motor alterations. The definition of consensus list and scales of clinical parameters presented in our study paves the path for a comprehensive clinical delineation of GRIN1-related disorders and by extension to GRD group, contributing to previous genotype-phenotype studies [6,9] and towards a precise definition of the natural history of these genetic conditions. Further, the novel inclusion of GRD-associated systemic dimension (including but not limited to gastrointestinal distress, sleep disturbance systemic functions symptomatologies) can provide discriminative clinical items to refine GRD delineation and stratification.

Functional annotation of *GRIN1* disease-associated variants is a crucial step towards the molecular identification of the pathophysiological mechanisms underlying GRD and, by extension, to define precision therapeutic strategies for GRD. The use of heterologous expression systems (*Xenopus laevis* oocytes, mammalian cell lines) is the current approach to functionally annotate *GRIN* variants. Despite synaptic microenvironment (metabolome, interactome, structural organisation) can have an impact on neuronal outcomes of *GRIN* variants, a growing number of data support a conservation of NMDAR mutations effects across cellular, ex vivo and patients data, especially for those variants affecting the extracellular and transmembrane domains [16]. This coincidence across cellular models can be different for *GRIN* variants affecting the intracellular domains (CTD) of the NMDAR, which are the object of a myriad of post-translational modifications (i.e., phosphorylation, glycosylation) and interaction with postsynaptic scaffolding proteins that regulate the turnover (surface density) and biophysical properties of the NMDAR.

The patients recruited in this study have been genetically diagnosed as patients harbouring *GRIN1* de novo missense mutations. The genetic diagnosis has been performed using whole exome sequencing. In agreement with the reported autosomal dominant inheritance pattern, the patients displayed the presence of a de novo *GRIN1* missense variant, located in different critical domains (defined as pathogenic variants hotspots; e.g., LBD and TMD vulnerability) strongly supporting the monogenic genetic aetiology of their neurodevelopmental alterations. Besides the presence of likely pathogenic *GRIN1* variants, the presence of additional genetic variants (i.e., affecting additional genes) might modulate (leveraging or worsening) the severity of GRD clinical manifestations. In most of the cases, *GRIN1* variant was the only genetic variant identified as "likely pathogenic", while (i) no additional genetic variants were detected, (ii) genetic variants were present, but with uncertain significance (VUS) or with an autosomal recessive inheritance pattern, (iii) inherited genetic variants (data not shown). Despite the genetic reports strongly confirm the autosomal dominant pattern and monogenic origin of the NDD, the potential role of additional genes (variants) cannot be ruled out.

In this study, we have identified paradigmatic *GRIN1* variants that affect the biogenesis and gating properties of the NMDAR. Surface expression studies showed that some precise amino acid substitutions and a TMD indel provoke a drastic reduction of NMDAR surface expression (depletion of mutant GluN1-SDD containing NMDARs in the cell surface), causing a LoF effect. Experimental data showed that the alteration of protein stability and/or oligomerisation/trafficking processes did not interfere with wildtype GluN1 subunit-containing NMDARs activity, and cause a relatively moderate clinical symptomatology, within the severity of GRD. Recently, our group has shown that heterozygous *GRIN1* protein truncating variants (PTVs), despite their inability to reach the plasma membrane, were not disease-associated [21]. This apparent clinical discordance between missense *GRIN1*-SDD missense and *GRIN1*-PTV might be explained by the Nonsense-Mediated mRNA Decay (NMD) pathway [22,23]. Indeed, NMD is a quality control pathway removing transcripts bearing premature termination codons (i.e., nonsense and frameshift mutations causing *GRIN1*-PTVs), which could avoid GluN1 truncated subunits synthesis and further dimerization/oligomerization, ultimately preventing *GRIN1*-PTV deleterious effects (observed in a patient with a homozygous *GRIN1*-Q556ter PTV; [9]). On the contrary, NMD pathway does not exert a quality control on missense mutations, and *GRIN1*-SDD subunits might form dimers and/or oligomers unable to traffic or dock to the cell surface, therefore reducing NMDAR surface density and provoking a net hypofunctionality and GRD clinical symptoms.

Beyond the surface trafficking defects of particular *GRIN1*-DNV, the functional annotation studies unveiled a disturbance of NMDAR channel properties in disease-associated non-SDD *GRIN1* missense variants. The functional stratification of these variants allowed to propose a hierarchical-based severity stratification, integrating *GRIN1* variants-dependent biophysical alterations of the NMDAR. Despite a relatively limited number of *GRIN1*-DNV characterised in our study, a relative phenotypic expressivity (mostly affecting epileptogenic activity, but not ID/DD phenotypes) and the potential effects of the developmental stage (age) and gender dimensions (do not considered in the present study, due to the limited cohort size), the integration of comprehensive functional and clinical data allowed retrieving a certain genotype-phenotype association. Indeed, the exclusive presence of GoF- or LoF-related alterations was associated with severe forms of GRD, while combined or complex effects (GoF and LoF-related NMDAR disturbance) were associated with less abundance of GRD severe symptoms. These data suggest that, rather than a dichotomic classification of *GRIN1*-DNV (LoF vs. GoF), *GRIN1* variants stratification paradigm must be revisited, considering the molecular pathophysiological continuum and the associated clinical spectrum.

## 4. Materials and Methods

### 4.1. Ethical Statement

Genetic report and clinical information from individuals harbouring *GRIN1* were provided by referring physicians. All legal guardians provided informed written consent for genetic testing in accordance with the Declaration of Helsinki and the protocol was approved by the local Ethics Committees of the participating study centers.

### 4.2. Plasmids

The expression plasmids for rat GluN1, GFP-GluN2A and GFP-GluN2B were kindly provided by Dr. Vicini [24]. Nucleotide changes for the production of *GRIN* variants were achieved by oligonucleotide-directed mutagenesis, using the QuickChange II XL site-directed mutagenesis kit according to the manufacturer’s instructions (Stratagene, San Diego, CA, USA), and verified by Sanger sequencing.

### 4.3. Cell Culture and Transfection

HEK-293T and COS-7 cell lines were obtained from the American Type Culture Collection and maintained at 37 °C in Dulbecco’s modified Eagle’s medium (DMEM), supplemented with 10% fetal calf serum and antibiotics (100 units/mL penicillin and 100 mg/mL streptomycin) and D-2-amino-5-phosphonopentanoic acid (D-AP5, Abcam, Cambridge, UK; 0.5–1 mM final concentrations, for HEK-293T and COS-7 cells, respectively) to avoid excitotoxicity. Transient expression of NMDARs in HEK-293T cells was achieved with polyethylenimine (PEI)-based transfection methods, and NMDAR-mediated currents were recorded 24 h after transfection. COS-7 cells were transfected with Lipofectamine^TM^ 2000 (Invitrogen, Watham, MA, USA) following the manufacturer’s instructions, and cells were fixed 24 h post-transfection for further immunofluorescence analysis. Cells were transfected with equimolar amounts of GluN1 and GFP-GluN2A/GluN2B subunits (1:1) for immunofluorescence experiments and using a 1:2 (GluN1:GluN2) ratio for electrophysiological recordings.

### 4.4. Immunofluorescence Analysis of NMDAR Surface Expression in COS-7 Cells

Transiently transfected COS-7 cells were washed in PBS and fixed with 4% paraformaldehyde. Surface expression of NMDARs was achieved by immunolabeling the extracellular GFP tag (GFP cloned in-frame within GluN2 subunits ATD) and incubating with anti-GFP antibody (Clontech, Kyoto, Japan) for 1 h at RT, under non-permeabilizing conditions. After washing, cells were incubated with anti-rabbit IgG-Alexa555 secondary antibodies (Life Technologies, Carlsbad, CA, USA), for 1 h at RT. The total amount of GFP-tagged GluN subunits was detected by the GFP endogenous fluorescent signal emitted by GFP-GluN2A/GluN2B constructs. Coverslips were mounted in ProLong antifade mounting medium (Life Technologies, Carlsbad, CA, USA) and images were acquired in a Nikon Eclipse 80i microscope (63 × /1.4 N.A. immersion oil objective).

### 4.5. Electrophysiological Recordings of NMDAR-Mediated Whole-Cell Currents in HEK-293T Cells

Electrophysiological recordings were obtained 24 h after transfection, perfusing the cells continuously with extracellular physiological bath solution (in mM): 140 NaCl, 5 KCl, 1 CaCl_2_, 10 glucose, and 10 HEPES, adjusted to pH 7.42 with NaOH. Glutamate (1 mM, Sigma-Aldrich, St. Louis, CA, USA) and glycine (50 μM; Tocris, Bristol, UK) were co-applied for 5 sec by piezoelectric translation (P-601.30; Physik Instrumente, Germany) of a theta-barrel application tool made from borosilicate glass (1.5 mm o.d.; Sutter Instruments, Novato, CA, USA) and the activated currents were recorded in the whole-cell configuration at a holding potential of −60 mV, acquired at 5 kHz and filtered at 2 kHz by means of Axopatch 200B amplifier, Digidata 1440A interface and pClamp10 software (Molecular Devices Corporation, San Jose, CA, USA). Electrodes with open-tip resistances of 2–4 MΩ were made from borosilicate glass (1.5 mm o.d., 0.86 mm i.d., Harvard Apparatus, Cambridge, MA, USA), pulled with a PC-10 vertical puller (Narishige, Japan) and filled with intracellular pipette solution containing (in mM): 140 CsCl, 5 EGTA, 4 Na_2_ATP, 0.1 Na_3_GTP and 10 HEPES, adjusted to pH 7.25 with CsOH. Glutamate and glycine-evoked currents were expressed as current density (-pA/pF; maximum current divided by input capacitance as measured from the amplifier settings) to avoid differences due to surface area in the recorded cells. The kinetics of deactivation and desensitization of the NMDAR responses were determined by fitting the glutamate/glycine-evoked responses at V_m_ −60 mV to a double-exponential function in order to determine the weighted time constant (τ_w,des_). 

### 4.6. Statistical Analysis

Comparison between experimental groups was evaluated using Prism9 (GraphPad Software, Inc., CA, USA), applying a One-Way Analysis of Variance (ANOVA) followed by a Bonferroni post-hoc test (cell surface expression experiments) or Dunn’s multiple comparison (electrophysiology experiments). For single pair comparisons, either Student’s *t* test (for parametric data) or Mann-Whitney U-test (for non-parametric data) were used. Data are presented as the mean ± SEM from at least three independent experiments.

### 4.7. Structural Models

The initial structures to construct the structural model for the triheteromeric NMDA receptor (GluN1)2-GluN2A-GluN2B were select according to (i) best resolution of the initial structures, (ii) structures covering the ATD, LBD and TM domains and (iii) triheteromeric structure to include GluN1, GluN2A and GluN2B subunits and its interfaces. 4PE5 [25] was used as the initial model for GluN1 and GluN2B subunits, while 5UOW (28232581) was used to for the GluN2A subunits and as the template for the assembly of the three interacting subunits. Modeller 9.20 [26] was used to model the lacking regions of the receptor and Scwrl4 [27] to position the non-determined side-chains. The initial model was energy-minimized using GROMACS 5 [28]. Variants GRIN1-R217W, -D227H, -S617C, -G620R, -M641V, -D732E, -P805L, -P805S, -A814D, -M818V, -G827R were introduced in the NMDA receptor final model using Pymol 2 (https://pymol.org/2/, accessed on 23 September 2021).

## Figures and Tables

**Table 1:** Comprehensive clinical assessment of *GRIN1*-DNV patients cohort. The table summarises the neurological symptoms associated with the presence of de novo *GRIN1* variants harboured by single individuals or, in certain cases (*GRIN1*-G620R, -D732E, -E834Q), in two familiarly unrelated individuals.

			SUPERIOR FUNCTIONS					MOTOR FUNCTION			
Clinical Case	Variant	Gender, Age	Intellectual Disability	Mood Disorders	Sleep Disorder	Verbal Communication	ASD Traits	Movement Disorders	Gait	Muscle Tone	Oculogyric Crisis
1	GRIN1-R548W	F, exitus (10 m.o.)	Severe	No	Yes (melatonin treatment)	No	No	Akinetic rigid syndrome, stereotypies	Non-acquired ambulation	Lower limb spasticity, axial hypotonia	N.A.
2	GRIN1-S617C	F; 9.5 years-old	Severe	Hyperactivity	Sleeps with the mother	<10 words	No	No	Motor delay, autonomous ambulation aquired at 3 y.o.	Normal	No
3	GRIN1-I619G620dup	F; 12 years-old	Severe	Irritability	No	No	No	No	Ambulation with double support	Hypotonia	No
4	GRIN1-G620R	M, 5 years-old	Severe	No	No	No. Language restricted to 5 purposeful sounds, understands easy orders		No	No ambulation. No sitting. Cephalic control	Severe axial hypotonia, hyperlaxity	No
5	GRIN1-G620R	F, 5.5 years-old	Severe	No	No	No. Unintelligible jargon, absence of purposeful language	Yes	Hyperkinesia, dyskinetic movements, hands-washing stereotypies	No ambulation. Sitting acquired at 4 y.o., standing with supports	Axial hypotonia, Upper limb hypertonia, hyperlaxity	N.A.
6	GRIN1-M641V	F, Exitus (20 y.o.)	Severe	Agressivity	No	No	Yes	No	No ambulation	Limbs spaticity axial hypotonia	
7	GRIN1-D732E	F, 11.5 years-old	Severe	Intolerance, frequent hanger behaviour, hypo- and hypersensitivity	No	Poor language (7 words, gestures, shouts) acquired at 4 years-old. Sporadic use od 2-3 bisyllabic words, and 3 bimodal signs	ASD traits, social interaction interest	Hands and swinging stereotypies; manual hyperkinesia	Walking acquired at 5 y.o., with external hand support, ability to walk and go up and down stairs	Axial hypotonia, lower limbs mild spactic signs, hyperlaxity	No
8	GRIN1-D732E	F, 18 years-old	Severe	No	No	No	No	Akinetic rigid syndrome, stereotypies	Autonomous ambulation in familiar spaces, unstability in uneven terrains	Upper limbs spasticity (lower limbs, difficult to assess)	No
9	GRIN1-P805S	F, 11.8 years-old	Severe	Hyperactivity, impulsiveness	No	No. Unintelligible jargon	No	No	Normal ambulation	Axial hypotonia, spasticity	No
10	GRIN1-P805L	M, 5 years-old	Severe	No	Difficulties falling asleep, multiple awakenings. (melatonin-tryptophane treatment)	No	No. Hand stereotypies	Oculomotor dsirturbances (oculogyric crisis starting at 4 m.o., visual tonic upgaze, horizontal nistagmus); dyskinetic movements	Intermitent cephalic control, no sitting, no ambulation	Spastic tetraparesis, axial hypotonia with limbs spasticity, generalized hypotonia	Yes
11	GRIN1-A814D	F, 11.8 years-old	Severe	Motor restlessness, paroxysmal agitation episodes	Difficulties falling asleep, awakenings; Resistant to neuroleptics, benzodiazepines, antihistaminic and melatonin	No	Yes	Dystonia and choreoathetosis	No ambulation, no cephalic control	Axial hypotonia	N.A.
12	GRIN1-M818V	M, 13.8 years-old	Moderate	No	Mild difficulties falling asleep, autolimitated	Yes. Difficulties in pragmatics	Yes	Stereotypies	Ambulation with motor clumsiness	Hypotonia	N.A.
13	GRIN1-G827R	M, 5.8 years-old	Severe	No	Yes	No	No	Akinetic rigid syndrome, stereotypies. Dystonic movements. Oculogyric crisis	Non-acquired	Lower limbs spasticity, axial hypotonia	Yes
14	GRIN1-E834Q	M, 14 years-old	Severe	Aggresivity, motor hyperkinesia	No	No	Yes	Stereotypies	Autonomous ambulation	Normal tone	No
15	GRIN1-E834Q	10 years-old	Severe	Disruptive, hyperkinetic and restlessness behaviour	No	No	No	Hyperkinesia, akathisia. Motor clumsiness, poor motor coordination, without ataxia or specific movement disorder	Autonomous ambulation acquired at 2.8 y.o.	Normal tone; Low axial tone until 1 y.o.	No
			**EPILEPSY**				**BRAIN IMAGING**			**G-I DISTRESS**	
**Clinical case**	**Variant**										
1	GRIN1-R548W		Neonatal myoclonia, tonic seizures and spasms. AED-controled				Asymmetric ventriculomegaly, thin corpus callosum and pulvinar nuclei			Dysphagia	
2	GRIN1-S617C		No				Normal			No	
3	GRIN1-I619G620dup		Absence seizures, onset at 18 m.o.				Prominence and rounding of the LVs frontal horns (Evans’ index= 0.35); Mega cisterna magna with prominence of the IV ventricle median aperture			No	
4	GRIN1-G620R		No				Macrocephaly; Reduced corpus callosum size, prominent ventricles and enlarged subdural space			Yes. Liquid dysphagia, gastroesophageal reflux, constipation, normal digestive endoscopy	
5	GRIN1-G620R		Oculocephalic crisis (onset: 5 m.o.) with remission. Valproate treatment stopped (4 y.o.)				Increased extra-axial spaces			No	
6	GRIN1-M641V		Absences, tonic seizures (period: 13 m.o to 13 y.o.)				Cortico-subcortical atrophy, corpus callosum hypoplasia			N.A.	
7	GRIN1-D732E		Single seizure episode (4 y.o.)				Normal (3 y.o.)			Oropharingeal dysphagia, constipation, episodic laxative treatment	
8	GRIN1-D732E		Non-febrile crisis (7 y.o.)				Normal			No	
9	GRIN1-P805S		No				Normal			No	
10	GRIN1-P805L		Yes, onset at 2 m.o. Tonic seizures. Clonic seizures and rigidity of lower limbs (frequency: 4-5 times/month). AED: Efficient carbamazepine and levetiracetam treatments				Normal			Yes, severe gastroesophageal reflux, gastric ulcers	
11	GRIN1-A814D		Epileptic encephalopathy (onset: 6 m.o.) with spasms and tonic seizures				Mild brain atrophy			Dysphagia, gastrostomy	
12	GRIN1-M818V		Focal epilepsy (onset: 2 y.o.) evolving to myoclonic seizures with reflex component (audiogenic). Topiramate efficacy. Absence of seizures since 3 y.o.				Normal			No	
13	GRIN1-G827R		Tonic seizures with laugh (onset: 3 m.o.) Absnece seizures with elevated gaze. AED efficacy				Increased extracerebral space with prominent sulci and ventricular size; reduced corpus callosum volume and thickness; decreased hippocampal size			No	
14	GRIN1-E834Q		No				Normal			No	
15	GRIN1-E834Q		Focal seizures with secondary generalised seizures (onset: 3 m.o.), evolving to night tonico-clonic seizures. Resistance to ketogenic diet, levociteram, clobazam, TPM combined to valproic acid. Seizures controled under valproic acid and LTG				Increased extra-axial space (diffuse brain atrophy)			No	

## Supplementary Materials

The following are available online at https://www.mdpi.com/article/10.3390/ijms222312656/s1.
