# Supplementary material for: Paradigmatic De Novo GRIN1 Variants Recapitulate Pathophysiological Mechanisms Underlying GRIN1-Related Disorder Clinical Spectrum
Source: Int J Mol Sci. 2021 Nov 23;22(23):12656. doi: 10.3390/ijms222312656 (PMC8657601; doi:10.3390/ijms222312656)
Supplement: Supplementary file 1 [file ijms-22-12656-s001.zip › ijms-1430181-supplementary.pdf]

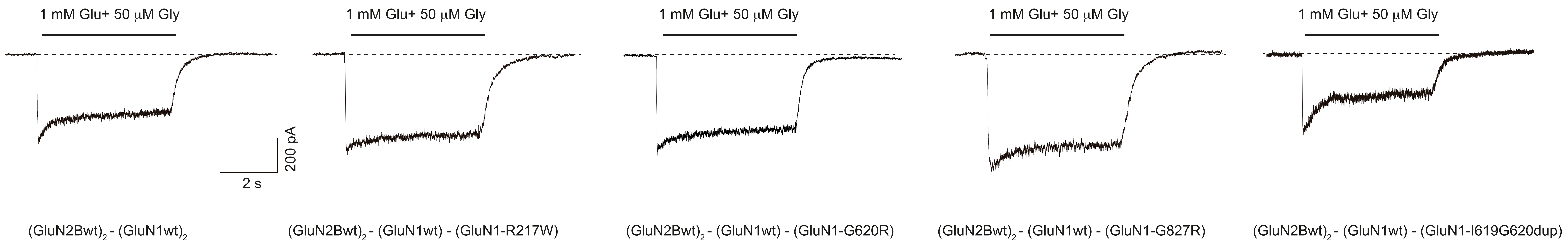

***GRIN* variant**	Ipeak (-pA)	Normalised current density (pA/pF)	Desensitization rate (msec)	Steady state (%)	Deactivation rate (msec)	Surface expression (%)
(GluN2B) 2 - (GluN1wt) 2	436.9 ± 126 (N=15)	36.38 ± 5.9 (N=14)	1.12 ± 0.3 (N=5)	62.77 ± 3.9 (N=13)	0.38 ± 0.04 (N=4)	100 ± 2.4 (N=81)
(GluN2B) 2 - (GluN1-R217W / wt)	213.8 ± 47.9 (N=15) ns	20.33 ± 5.2 (N=16) ns	3.168 ± 1.4 (N=5) ns	72.48 ± 2.9 (N=16) ns	0.5243 ± 0.05 (N=7) ns	88.34 ± 4.422 (N=25) ns
(GluN2B) 2 - (GluN1-I619G620dup / wt)	597.3 ± 244.5 (N=7) ns	56.96 ± 24.4 (N=7) ns	0.80 ± 0.4 (N=2) ns	69.57 ± 2.5 (N=4) ns	0.51 ± 0.09 (N=4) ns	106.5 ± 6.224 (N=23) ns
(GluN2B) 2 - (GluN1-G620R / wt)	269.3 ± 63.6 (N=8) ns	23.61 ± 3.5 (N=8) ns	N.A.	N.A.	N.A.	N.A.
(GluN2B) 2 - (GluN1-G827R / wt)	401 ± 104 (N=5) ns	43.8 ± 20.7 (N=5) ns	0.81 ± 0.09 (N=2) ns	**81.6 ± 2.1 (N=5) *****	0.48 ± 0.05 (N=2) ns	78.95 ± 4.78 (N=24) ns

Supplementary Figure S1: Electrophysiological characterisation of NMDAR-mediated currents in HEK-293T heterologously expressing disease-associated GRIN1-DNVs
